# Revision of *Macrima* Baly, 1878 (Coleoptera: Chrysomelidae: Galerucinae)

**DOI:** 10.3390/insects16070685

**Published:** 2025-06-30

**Authors:** Chuan Feng, Xingke Yang, Jan Bezděk, Siqin Ge

**Affiliations:** 1 State Key Laboratory of Animal Biodiversity Conservation and Integrated Pest Management, Institute of Zoology, Chinese Academy of Sciences, Beijing 100101, China; fengchuan23@ioz.ac.cn (C.F.); yangxk@ioz.ac.cn (X.Y.); 2 University of Chinese Academy of Sciences, Beijing 100049, China; 3 Department of Zoology, Fisheries, Hydrobiology and Apiculture, Mendel University in Brno, Zemědělská 1, 61300 Brno, Czech Republic

**Keywords:** leaf beetles, new synonymy, taxonomy, type specimen

## Abstract

This study presents a taxonomic revision of the genus *Macrima* Baly, 1878 to advance the understanding of suprageneric, non-monophyletic “Monoleptites”. Through comprehensive morphological examinations of all type specimens, including detailed analyses of male and female genitalia and the structure of the cavitous anterior part of the head, we propose *M. roseofulva* Medvedev, 2011 and *M. ferrugina* Jiang, 1990 as new synonyms of *M. aurantiaca* (Laboissière, 1936), *M. bifida* Yang, 1992 a new synonym of *M. cornuta* (Laboissière, 1936), and *M. yunnanensis* (Laboissière, 1936) as a new synonym of *M. pallida* (Laboissière, 1936). In addition, *M. costatipennis* Jacoby, 1896 is transferred to *Hoplosaenidea* Laboissière, 1933 (comb. nov.). Color photos of habitus, body details, and genitalia are presented for all species.

## Simple Summary

The genus *Macrima* belongs to the subfamily Galerucinae and is widely distributed in the Oriental Region. In this study, all species of the genus *Macrima* were revised based on type specimens. In total, *Macrima* comprises seven species worldwide. A key to all *Macrima* species is provided.

## 1. Introduction

The subfamily Galerucinae includes three supertribes: Alticitae, Galerucitae, and Serraticollitae [1]. Galerucitae is one of the most diverse groups of leaf beetles, encompassing 7145 species across 543 genera worldwide [2]. There are 127 genera and 1213 species recorded in China [1,3]. Among the Galerucitae, the section “Monoleptites” is highly diverse, characterized by the distinctly elongated first tarsomere of the hind-legs. The section “Monoleptites” is a suprageneric, non-monophyletic name and a rank that was established by Chapuis [4], and it is generally accepted to describe a highly diverse group of Galerucitae in the tribe Luperini [5,6,7,8].

Genus *Macrima* Baly, 1878 belongs to “Monoleptites”, with various structural modifications on the frons. *Macrima* was established by Baly for *Macrima armata* Baly, 1878 (type species) from Kashmir [9]. In the original description, *Macrima* was compared only with the genus *Aulacophora* Chevrolat, 1836. This probably led Jacoby [10,11,12,13,14,15] to misconceive the genus *Macrima*, as he successively described nine Oriental and even one African species, which were almost all subsequently transferred to the genera *Hoplosaenidea* Laboissière, 1933 and *Polexima* Weise, 1903, respectively [16,17,18,19,20,21,22,23]. The last remaining, *Macrima costatipennis* Jacoby, 1896, is transferred to *Hoplosaenidea* in the present paper. Fairmaire [24] and Weise [25] were probably unaware of Baly’s description of the genus *Macrima*. In the same year, they described the genera *Sepharia* Fairmaire, 1889, and *Glechonis* Weise, 1889, whose type species, *Sepharia rubricata* Fairmaire, 1889 and *Glechonis rubripennis* Weise, 1889, are synonyms [26]. Formally, *Sepharia* and *Glechonis* were synonymized with *Macrima* by Gressitt and Kimoto [27].

Due to above-mentioned situation, most species of the genus *Macrima* were originally described as *Sepharia*. Apart from the already mentioned *Macrima armata* and *M. rubricata*, Jacoby [28] described *Sepharia frontalis* Jacoby, 1890 which was synonymized with *Macrima armata* by Maulik [29]. An additional four new species were added by Laboissière [30] and one by Ogloblin [31]. Recently, the last four species, already known as *Macrima*, were described by Jiang [32], Yang [33], and Medvedev [34,35].

## 2. Materials and Methods

The morphological characters were examined using a Leica S8AP0 microscope. The male and female genitalia of each species were dissected using the following procedure [36,37]: for dried or ethanol-preserved specimens, the abdomen was carefully removed from each specimen, bathed in boiling water for 5–10 min, then transferred to a vial containing 10% KOH solution and bathed for 3–5 min. The abdomen with the male or female genitalia was washed in distilled water 3 to 4 times, then transferred to a concave slide, and the genitalia were separated from the abdomen using fine forceps and a hooked dissecting pin. The genitalia were mounted in a drop of glycerol on slides for photography. The terminology of male and female genitalia follows [7]. The term aedeagus refers to the entire male copulatory organ, i.e., median lobe (=penis), dorsal process, and tegmen [38]. Images of the habitus and genitalia were taken by using a Canon EOS R5 digital camera (IZCAS, Beijing, China). To obtain the full depth of focus, all images were stacked using HELICON FOCUS 7.7.4, and the resulting output was edited with Adobe Photoshop CC 2018.

Labels written in Chinese are translated into English and cited verbatim. The abbreviations used in this study are type locality (TL), type deposition (TD). The materials in this study are deposited in the following institutions: Institute of Zoology, Chinese Academy of Sciences, Beijing, China (Kuiyan Zhang) (IZCAS). Jan Bezděk collection, Brno, Czech Republic (JBCB). Museum of Comparative Zoology, Harvard University, Cambridge, MA, USA (Crystal Maier) (MCZ). Muséum National d’histoire Naturelle, Paris, France (Antoine Mantilleri) (MNHN). Museo Civico di Storia Naturale “Giacomo Doria”, Genova, Italy (Roberto Poggi) (MSNG). Natural History Museum, London, UK (Michael Geiser) (NHMUK). Naturkundemuseum, Erfurt, Germany (Matthias Hartmann) (NMEG). National Museum, Prague, Czech Republic (Lukáš Sekerka) (NMPC). Naturhistorisches Museum, Wien, Austria (Matthias Seidel) (NMW). Royal Belgian Institute of Natural Sciences, Brussels, Belgium (Pol Limbourg) (RBNS). Shanghai Entomological Museum, Shanghai, China (Li Dai) (SEM). Zoological Institute of the Russian Academy of Sciences, Russia (Alexey G. Moseyko) (ZIN). Zoological Museum Hamburg, Hamburg, Germany (Jan-Henrik Pamin) (ZMH).

## 3. Results

### 3.1. Taxonomy

*Macrima* Baly, 1878: 377 [9]. Type species: *Macrima armata* Baly, 1878, by original designation.

*Sepharia* Fairmaire, 1889: 78 [24]. Type species: *Sepharia rubricata* Fairmaire, 1889, designated by Laboissière, 1936. Synonymized by Gressitt and Kimoto, 1963: 650 [27].

*Glechonis* Weise, 1889: 130 [25]. Type species: *Glechonis rubripennis* Weise, 1889, by monotypy. Synonymized by Gressitt and Kimoto, 1963: 650 [27].

Distribution Oriental region.

### 3.2. Species Descriptions

#### 3.2.1. *Macrima armata* Baly, 1878 

*Macrima armata* Baly, 1878: 377 [9]. TL: Jhelam Valley [Azad Kashmir, Pakistan]. TD: NHMUK.

*Sepharia frontalis* Jacoby, 1890: 254 [28]. Synonymized by Maulik, 1936: 561 [29]. TL: Kashmir. TD: MCZ, NHMUK, RBNS.

Distribution. China (Xizang), India (Jammu and Kashmir, Himachal Pradesh, Sikkim), Nepal, and Pakistan.

Type specimens examined. *Macrima armata*: not examined. *Sepharia frontalis*: 1♂, Syntype (NHMUK), “Type//Kashm.//Jacoby Coll. 1909-28a//Sepharia frontalis Jac.”; 1♀, Syntype (MCZ), “2nd Jacoby Coll.//*frontalis* Jac.//Type 18294”. 1♀, Syntype (RBNS), “Kashmir Coll. Jacoby//Collect. Duvivier//M. Jacoby det.: Sepharia frontalis Jac//Ex-typis//cf. Entomolog. XXIII, 1890, p. 254”.

Other specimens examined. 1♂, India, Himachal Pradesh, Jibhi, Seraj, May 1926, H. G. Champion leg., NHMUK. 1♀, India, Himachal Pradesh, Kulu valley, Naggar, 15 May 1997, A. Stauder leg., NMW. 3♂♂, Nepal, Karnali, Humla, 6 km NW of Simikot, Dandaphaya Dharapuri., 30°00′09″ N 81°46′08″ E, 2300 m, 18 June 2001, A. Kopetz leg., NMEG. 7♂♂, Nepal, Karnali, Humla, 14 km NW of Simikot, Kermi, 30°02′55″ N 81°42′20″ E, 2800 m, 19 June 2001, A. Kopetz leg., NMEG. 1♂1♀, Nepal, Karnali, Humla, 18 km NW of Simikot, Chumsa Khola bridge., 30°22′25″ N 81°39′06″ E, 2950 m, 20–22 June 2001, A. Kopetz leg., NMEG. 2♂♂, Nepal, Karnali, Humla, 5 km SE of Simikot, NE of Chhipra, Chuwa Khola, 29°56′33″ N 81°51′24″ E, riverside, 2200 m, 9 July 2001, A. Weigel leg., NMEG. 1♂, Nepal, Karnali, Humla, Simikot, 29°58′25″ N 81°49′07″ E, 3100 m, 10 July 2001, A. Weigel leg., NMEG. 2♂♂, Nepal, Karnali, Humla, 2 km SE of Simikot, Humla Karnali, 29°57′44″ N 81°50′21″ E, 2250 m, 16 June 2022, A. Weigel leg., NMEG. 1♀, Nepal, Karnali, Mugu, 31–35 km N of Jumla, 29°35′24″ N 82°08′45″ E–29°33′32″ N 82°09′30″ E, 1740–2600 m, 27 June 2022, A. Weigel leg., NMEG. 1♂, Pakistan, Kaghan-Tal, Balakot, ca 1000 m, 7–8 July 2003, Heinz leg., NMEG. 1♂, Pakistan, Kaghan-Tal, Balakot, ca 1000 m, 21–24 July 2003, Heinz leg., NMEG. 9♂♂, Pakistan, Swat, Madyan, 35°70′ N 71°90′ E, 1400 m, 19 June–4 July 1971, C. Holzschuh leg., NMEG.

**Figure 1:**
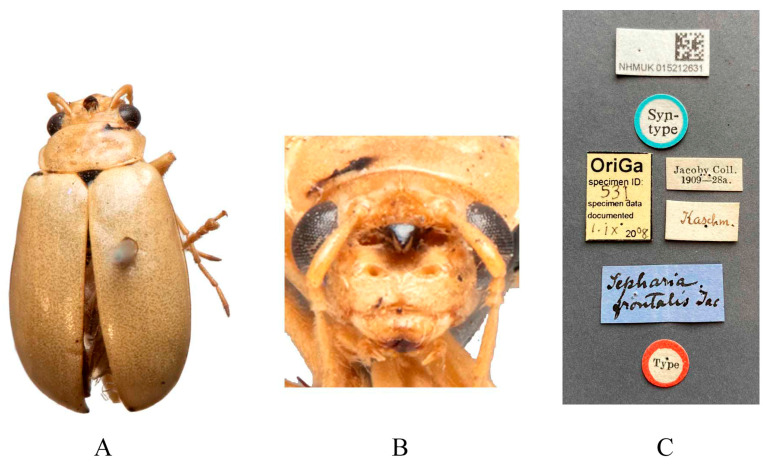
Syntype of *Sepharia frontalis* Jacoby, 1890. (**A**) Habitus, dorsal view; (**B**) habitus, head view of the male; (**C**) label (primary label, type label, repository label).

**Figure 2:**
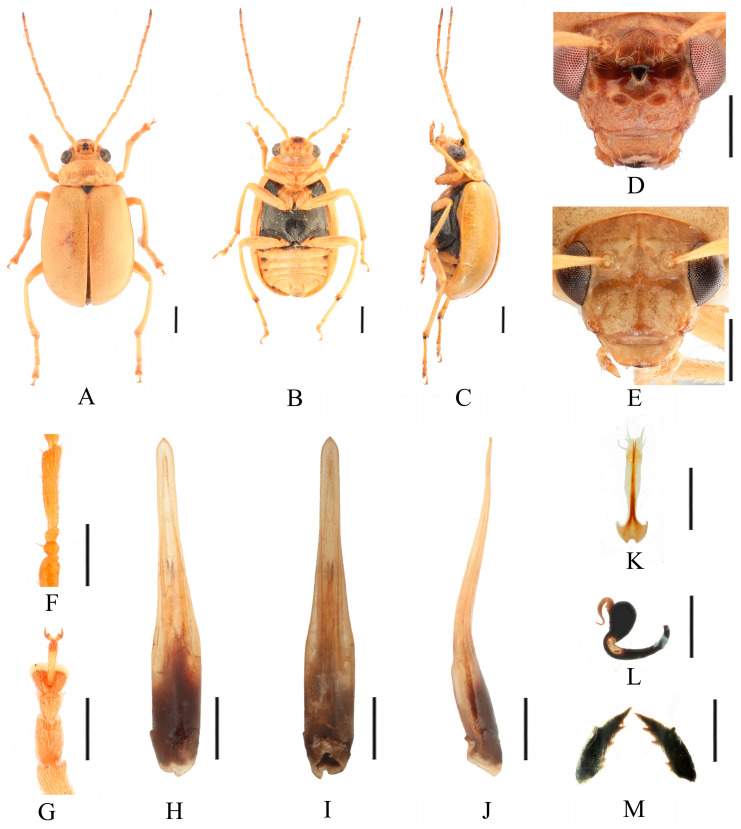
*Macrima armata* Baly, 1878. (**A**) Habitus, dorsal view; (**B**) habitus, ventral view; (**C**) habitus, lateral view; (**D**) habitus, head view of the male; (**E**) habitus, head view of the female; (**F**) antennomeres II–IV; (**G**) tarsomeres of front leg; (**H**) aedeagus, dorsal view; (**I**) aedeagus, ventral view; (**J**) aedeagus, lateral view; (**K**) vaginal palps; (**L**) spermatheca; (**M**) bursa sclerites. Scale bars: 1 mm (**A**–**C**); 0.5 mm (**D**–**M**).

Redescription (Figure 1 and Figure 2). Male: length: 7.6–8.4 mm, width: 3.8–4.1 mm. General color yellow, scutellum, basal part of inner margin of epipleuron, apex of suture, lateral of mesosternum, metasternum, and base of first metatarsomere black, ventral surface of head with a black spot, prosternum with three black spots, abdominal ventrites 1–5 with black spots on each side, pygidium with a black spot at apex. Vertex with sparse punctures, median process pointed on apex, lower edge of frons with two round cavities. Antennae slender, equal to length of body. First antennomere shiny bare, rod-shaped, second to eleventh antennomeres with short hairs, second antennomere equal to third (Figure 2F), fourth antennomere about 5.2× as long as third, fifth to eleventh antennomeres shorter than fourth and gradually shortened. Pronotum about 1.8× as wide as long, lateral margins slightly curved, basal margin slightly convex, apical margin slightly concave, disc with one pair of shallow impressions. Scutellum triangular, smooth, impunctate. Elytra wider than pronotum, humeri convex, disc with dense, minute punctures. Elytral epipleuron broad at base, continues towards apex. Legs thin, densely covered by short recumbent setae. First tarsomeres of front legs strongly swollen and flattened (Figure 2G). Each tibia with one spur at apex. Aedeagus slender, with parallel sided at base, gradually narrowed at middle, and middle to apex with parallel sided, apex pointed, slightly curved at middle in lateral view (Figure 2J). Female: length: 7.4–7.6 mm, width: 3.7–4.0 mm. Elytra red or reddish-brown, ventrites 1–3 or 1–5 with black spots on each side, or ventrites 1–3 with black transverse strip. Anterior part of head (Figure 2E) with a transverse depression and a longitudinal depression. First tarsomeres of front legs slender. Spermatheca (Figure 2L) with big and rounded nodulus, middle part short, and cornu slightly curved. Vaginal palps (Figure 2K) with wide base and pointed apex, each palp with three setae placed at apex, additional five setae subapically. Bursa sclerites sharp with 4–6 teeth at inner side (Figure 2M).

Differential diagnosis. Males of *Macrima armata* Baly, 1878 are distinguished by the distinct black frontal process (Figure 2D). This character is unique within the genus; no other species possesses a black frontal process. Females of this species can be distinguished by a cruciform depression on the frons (Figure 2E).

Comments. Baly (1878) did not state the exact number of specimens available for the description, but he explicitly described both males and females from Jhelum Valley (Azad Kashmir, Pakistan). Baly’s collection, now deposited in NHMUK, contains three specimens identified as *Macrima armata*. Only one of these specimens (female) bears the locality label Jhelum Valley and was subsequently provided with the characteristic round type label. Maulik (1936) mentioned only the female as the type specimen, and it was probably he who attached the type label. However, in our opinion, this specimen is a female of *Macrima pallida*. Additional two specimens are a male of *M. armata* from Kullu (Himachal Pradesh, India) and a female from Mussoorie (Utarakhand, India), which again pertains to *M. pallida*. The locality data of these two specimens do not correspond to the original description and therefore cannot be considered type specimens of *M. armata*. Baly included a male in his original description; however, it is unclear whether this male has not survived or whether Baly made a mistake in his description and simply forgot to include the Kullu locality in the description. Baly explicitly stated that the frontal process in the male is black, which corresponds exactly to the current concept of the species, since *M. armata* is the only species in which the frontal process is black.

#### 3.2.2. *Macrima aurantiaca* (Laboissière, 1936) 

*Sepharia aurantiaca* Laboissière, 1936: 250 [30]. TL: Yunnan: Pe Yen Tsin. TD: ZMH, RBNS.

*Macrima aurantiaca*: Gressitt and Kimoto, 1963: 652 [27].

*Macrima ferrugina* Jiang, 1990: 138 [32]. Syn. nov. TL: Xizang: Modog. TD: IZCAS, SEM.

*Macrima roseofulva* Medvedev, 2011: 251 [35]. Syn. nov. TL: Nepal, Koshi, 3 km E Dharan. TD: ZIN.

Distribution. Bhutan, China (Guangxi, Sichuan, Yunnan, Xizang), India (Sikkim), Nepal.

Type specimens examined. *Sepharia aurantiaca*: 1♂, Syntype (ZMH), “Pe Yen Tsin, Yunnan, ex de Touzalin//TYPE//Sepharia aurantiaca m. V. Laboissière—Dét.//Le Moult vend. via Reinbek Eing Nr. 1, 1957//ZMH 844373”; 1♀, Syntype (ZMH), “Yunnan//TYPE ♀//Sepharia aurantiaca m. V. Laboissière—Dét.//Le Moult vend. via Reinbek Eing Nr. 1, 1957//ZMH 844374”; 1♀, Syntype (RBNS), “Coll. R. I. Sc. N. B., Chine, PE YEN TSIN, YUNNAN, Coll. de. Touzalin//Sepharia aurantiaca 1936 m. V. Laboissière—Dét.//Para- type”. *Macrima ferrugina*: ♂, Holotype (SEM), “China, Xizang, Motuo//9 September 1979, Gentao Jin & Jianyi Wu leg.//HOLOTYPE//24202238//TYPE Macrima ferrugina sp. nov.”; 1♂, Paratype (SEM), “China, Xizang, Motuo//9 September 1979, Gentao Jin & Jianyi Wu leg.//PARATYPE//24202239//PARATYPE Macrima ferrugina sp. nov.”; 1♀, Paratype (IZCAS), “China, Xizang, Motuo, Bangxin//1300 m a. s. l., 1 December 1982, Yinheng Han leg.//ALLOTYPE”. *Macrima roseofulva*: ♂, Holotype (ZIN), “Nepal, Koshi, 3 km N Dhankuta, 18.VII.1995, O. Gorbunov leg.//HOLOTYPUS Macrima roseofulva L. Medvedev”.

Other specimens examined. 1♂, China, Guangxi province, Tianlin, Linaoshan, 1300–1400 m a. s. l., 28 May 2002, Jianwen Liu leg., IZCAS. 2♂♂, China, Sichuan, Liangshan Mts., Xichang, 2700 m, 2 July 2002, S. Murzin & I. Shokhin leg., NHMUK. 1♂, China, Yunnan province, Lijiang, 2300 m a. s. l., 10 July 1984, Shuyong Wang leg., IZCAS. 2♂♂, China, Yunnan province, Zhongdian, Chongjianghe, 2300 m a. s. l., 4 August 1984, Dajun Liu leg., IZCAS. 1♂, China, Yunnan, 14 km SE of Tengchong, Renjiafen, 24°56.0-5′ N 98°35.0-7′ E, 2025–2145 m, 4 July 2016, J. Hájek & J. Růžička leg., NMPC. 2♀♀, China, Xizang, Motuo, Xirang, 750 m a. s. l., 7 November 1998, Jian Yao leg., IZCAS. 2♀♀, China, Xizang, Motuo, Madi, 1000 m a. s. l., 14 November 1998, Jian Yao leg., IZCAS. 1♂, China, Xizang, Motuo, Didong, 800 m a. s. l., 3 November 1998, Jian Yao leg., IZCAS. 2♀♀, China, Xizang, N of Brahmaputra great bend, 30°00′-07′ N 94°52′–95°09′ E, 2050–2400 m, L. & R. Businský leg., NMW. 1♂, China, Xizang, Tsangpo Gorge, Gompo Ne, 24 November 1924, F. Kingdon Ward leg., NHMUK. 1♀, India, Sikkim, Gantok, Fambong-lho forest, 2000–2500 m, 8–15 July 1997, J. Schneider leg., JBCB. 1♂, Nepal, Karnali, Humla, 20 km W of Simikot, 2 km SE of Chala, 29°58′49″ N 81°39′30″ E, 3500 m, juniperus meadow—coniferous wood, 27–28 June 2001, A. Kopetz leg., NMEG. 1♂, Nepal, Karnali, Mugu, Mugu Karnali Taka, 29°34.43′ N 82°23.54′ E, 2200 m, 29 June 1999, M. Hartmann leg., NMEG. 1♂, Nepal, Khesewa, 27°22′30″ N 87°48′54″ E, 1900–2200 m, deciduous forest, 25.v.2003, A. Weigel leg., NMEG. Bagmati, Kathmandu valley NW of Kathmandu, near Tinpiple, 27°46′12″ N 85°16′18″ E, 1450 m, 1 June 2013, 1♂, J. Küssner leg., NMEG. 1♂, Nepal, Manaslu Mts., E slope, Machhakhola valley, Gumda to Lapsibot, 28°11′49″ N 84°50′07″ E, 1500–1900 m, 23.v.2006, J. Schmidt leg., NMEG. 1♂, Nepal, Helambu, S slope Ganja La, Dukpu Kharka, 4000 m, 6–7 August 1998, C. Berndt leg., NMEG. 2♂♂, Nepal, Chandrakot, 25 km NW of Pokhara, 18 June 1999, Z. Andrš leg., JBCB. 1♂, Nepal, Arun valley, Mongmaya-Sultibari, 400–500 m, 2 June 1988, Lebisch & Probst leg., JBCB. 1♂, Nepal, Karnali, Humla, ca 41 km E of Simikot, Chauthala, 29°41′47″ N 82°06′04″ E, 2620 m, 23 June 2002, A. Kopetz leg., JBCB. 1♂, Nepal, Karnali, Humla, 21 km SE to 28 km E of Simikot, 29°49′49″ N 81°58′21″ E to 29°47′29″ N 82°01′15″ E, 2530–3200 m, 21 June 2022, A. Kopetz leg., JBCB. 1♂, Nepal, Karnali, Jumla, 9 km N of Jumla, Khali Chaur, 29°21′28″ N 82°09′32″ E, 3540 m, 1 July 2022, A. Weigel leg., NMEG. 1♂, Nepal, Annapurna Mts., Banthanti, Ghorapani to Ulleri., 2300 m, 13 September 2003, Schmidt leg., NMEG.

**Figure 3:**
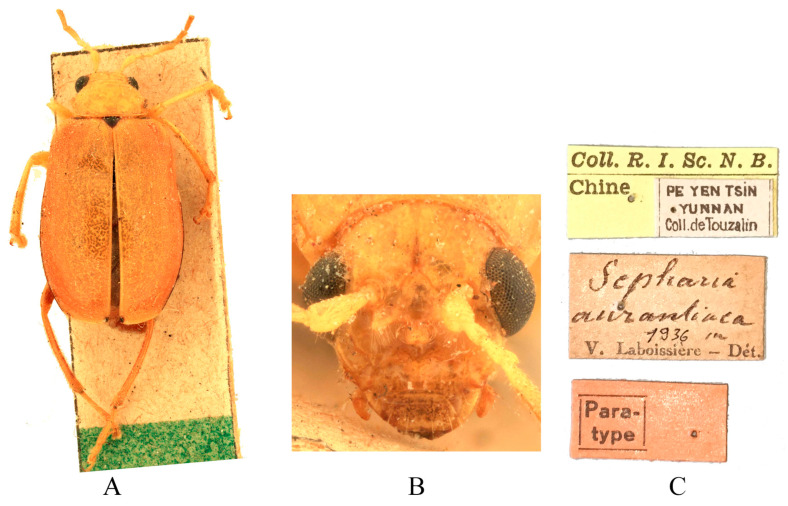
Syntype of *Sepharia aurantiaca* Laboissière, 1936. (**A**) Habitus, dorsal view; (**B**) habitus, head view of the male; (**C**) label (primary label, type label, repository label).

**Figure 4:**
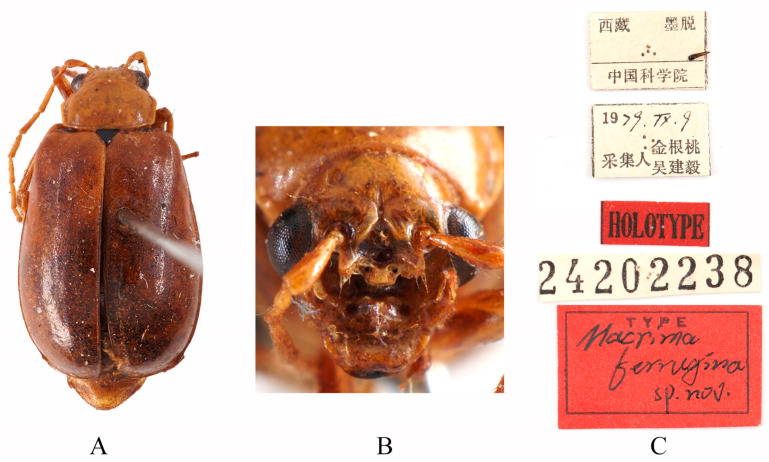
Holotype of *Macrima ferrugina* Jiang, 1990. (**A**) Habitus, dorsal view; (**B**) habitus, head view of the male; (**C**) label (primary label, type label, repository label).

**Figure 5:**
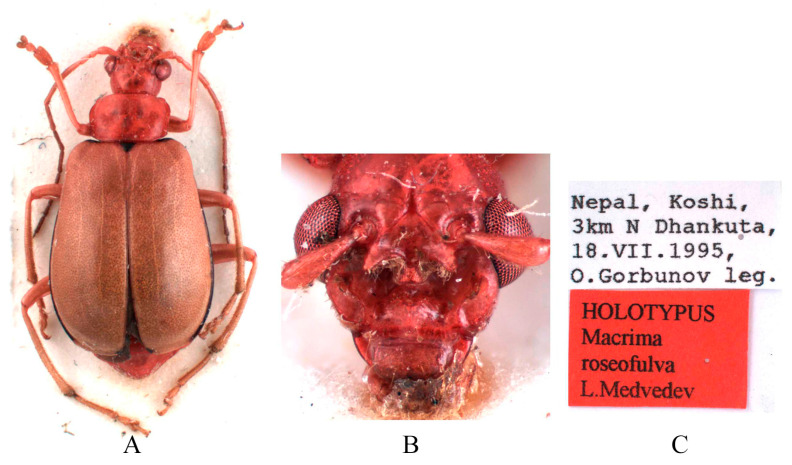
Holotype of *Macrima roseofulva* Medvedev, 2011. (**A**) Habitus, dorsal view; (**B**) habitus, head view of the male; (**C**) label (primary label, type label, repository label).

**Figure 6:**
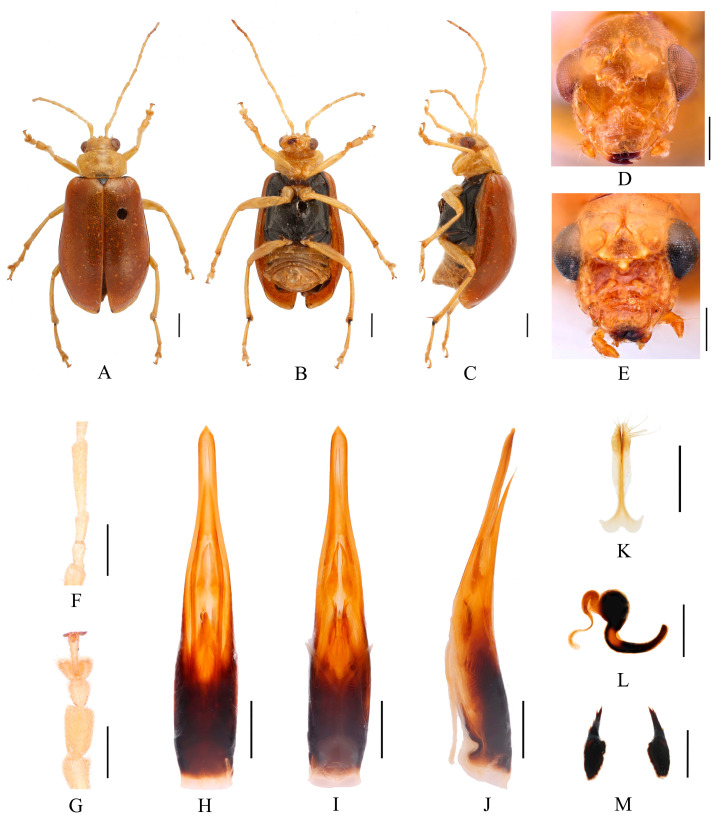
*Macrima aurantiaca* (Laboissière, 1936). (**A**) Habitus, dorsal view; (**B**) habitus, ventral view; (**C**) habitus, lateral view; (**D**) habitus, head view of the male; (**E**) habitus, head view of the female; (**F**) antennomeres II–IV; (**G**) tarsomeres of front leg; (**H**) aedeagus, dorsal view; (**I**) aedeagus, ventral view; (**J**) aedeagus, lateral view; (**K**) vaginal palps; (**L**) spermatheca; (**M**) bursa sclerites. Scale bars: 1 mm (**A**–**C**); 0.5 mm (**D**–**M**).

Redescription (Figure 3, Figure 4, Figure 5 and Figure 6). Male: length: 7.2–8.0 mm, width: 3.7–3.8 mm. General color yellow or yellowish-brown, scutellum, basal part of inner margin of epipleuron, apex of suture, lateral of mesosternum, metasternum and base of first metatarsomere black, ventral surface of head with a black spot, prosternum with three black spots, abdominal ventrites 1–5 with black transverse strip, pygidium with a black spot at apex. In some specimens, elytra red or reddish-brown, lateral margins of elytra black, ventrites 1–5 with black spots on each side, or ventrites 1–3 with black transverse strip, ventrites 4–5 with black spots on each side. Vertex with sparse punctures, upper edge of frons adorned between the antennae with two small processes, median process rounded on apex and pubescent below, lower edge of frons with two triangular processes and two round cavities, lateral edges of frons each with one triangular process. Antennae slender, equal to length of body. First antennomere shiny bare, rod-shaped, second to eleventh antennomeres with short hairs, second antennomere shortest, third antennomere 1.25× as long as second (Figure 6F), fourth antennomere about 2.6× as long as third, fifth to eleventh antennomeres shorter than fourth and gradually shortened. Pronotum about 1.65× as wide as long, lateral margins slightly curved, basal margin slightly convex, apical margin slightly concave, disc with one pair of shallow impressions. Scutellum triangular, smooth, impunctate. Elytra wider than pronotum, humeri convex, disc with dense, minute punctures. Elytral epipleuron broad at base, continues towards apex. Legs thin, densely covered by short recumbent setae. Fore tibia without spur at apex, first tarsomeres of front legs strongly swollen and flattened (Figure 6G), 2.6× as long as wide. Aedeagus slender, with parallel sided at base, gradually narrowed towards subapex, subapex to apex with parallel sided, apex pointed, slightly curved at middle in lateral view (Figure 6J). Female: length: 8.6–8.8 mm, width: 4.0–4.5 mm. Elytra red or reddish-brown, ventrites 1–3 or 1–5 with black spots on each side, or ventrites 1–3 with black transverse strip. Upper edge of frons adorned between the antennae with two small processes, median process small and rounded on apex, lower edge of frons with two small processes, and a longitudinal ridge in middle (Figure 6E). Fore tibia without spur at apex, first tarsomeres of front legs slender. Spermatheca (Figure 6L) with big and rounded nodulus, middle part short, and cornu slightly curved. Vaginal palps (Figure 6K) with wide base and pointed apex, each palp with three setae placed at apex, additional five setae subapically. Bursa sclerites sharp with 5–6 teeth at outside (Figure 6M).

Differential diagnosis. This species closely resembles *Macrima hartmanni* Medvedev, 2009 in anterior part of head. But it differs in having two large triangular processes on upper part of frons (Figure 6D), whereas *M. hartmanni* has only two small, blunt processes. Females of this species can be distinguished by three small rounded protuberances positioned above the middle of frons, whereas *M. hartmanni* has a single elongated median process.

Comments. We examined type specimens of *M. aurantiaca* deposited in ZMH (1 male, 1 female) (Figure 3) and RBNS (1 female) along with the type specimens of *M. ferrugina* from SEM (2 males) (Figure 4) and IZCAS (1 female), as well as the holotype of *M. roseofulva* (1 male) (Figure 5) deposited in ZIN. Both *M. roseofulva* and *M. aurantiaca* exhibit identical frontal modifications. Although the lower edge of the frons in *M. ferrugina* has two rounded cavities (Figure 4B), its aedeagus is morphologically indistinguishable from that of *M. aurantiaca*. Based on these morphological congruencies, we propose *M. roseofulva* and *M. ferrugina* as new synonyms of *M. aurantiaca*.

#### 3.2.3. *Macrima cornuta* (Laboissière, 1936) 

*Sepharia cornuta* Laboissière, 1936: 250 [30]. TL: Sze-Tchouen: Tchoug-King; env. de Ta-Tsien-Lou, Mo-Si-Mien. TD: MNHN, RBNS, ZMH.

*Macrima cornuta*: Gressitt and Kimoto, 1963: 652 [27].

*Macrima bifida* Yang, 1992: 258 [33]. Syn. nov. TL: Songpan County (32.6° N, 103.6° E), Sichuan Province. TD: IZCAS.

Distribution. Bhutan, China (Gansu, Ningxia, Sichuan, Xizang, Yunnan).

Type specimens examined. *Sepharia cornuta*: 1♂, Syntype (RBNS), “Tchoug-King Sze-Tchouan//Sepharia cornuta m. V. Laboissière—Dét.//TYPE//Ex-Typis//cf. Ann. Soc. Ent. Fr., CV, 1936, p. 250, fig. 49//R. Mus. Hist. Nat. Belg. I. G. 12.752”; 1♀, Syntype (ZMH), “TYPE ♀//Tchoug-King Sze-Tchouan//Sepharia cornuta m. V. Laboissière—Dét.//ZMH 844375”; 1♀, Syntype (MNHN), “Museum Paris, Se-Tchouen, env. de Ta-Tsien-Lou, Mo-Sy-Mien, Père Aubert 1902//TYPE ♀//Sepharia cornuta m. V. Laboissière—Dét.”. *Macrima bifida*: 1♂, Holotype (IZCAS), “Sichuan Songpan//4 July 1990, Jupeng Liu leg.//HOLOTYPE//Macrima bifida sp. nov. Yang”.

Other specimens examined. 2♂♂, China, Gansu province, Wen country, 2300 m a. s. l., 18 July 1986, Hongjian Wang leg., IZCAS. 4♂♂, China, Gansu province, Zhouqu, Shatanlinchang, 2400m a. s. l., 14 July 1999, Jian Yao leg., IZCAS. 3♂♂, China, Gansu province, Zhouqu, Shatanlinchang, 2400m a. s. l., 14 July 1999, Tongli He leg., IZCAS. 3♂♂, China, Gansu province, Zhouqu, Shatanlinchang, 2400 m a. s. l., 16 July 1999, Jian Yao leg., IZCAS. 1♀, China, Sichuan province, Chengdu, 20 June 1938, IZCAS. 1♂3♀♀, China, Sichuan province, Mt Emei, Baoguosi, 550–750 m a. s. l., 12 July 1957, Youcai Yu leg., IZCAS. 8♀♀, China, Sichuan province, Luding, Moxi, Hailuogou, 1420 m a. s. l., 18 September 1982, Shuyong Wang leg., IZCAS. 3♂♂9♀♀, China, Sichuan province, Luding, Moxi, 1500 m a. s. l., 14 September 1982, Shuyong Wang leg., IZCAS. 2♀♀, China, Sichuan province, Luding, Moxi, Hailuogou, 1900 m a. s. l., 11 August 2004, Xia Wan leg., IZCAS. 4♂♂, China, Sichuan province, Luding, Moxi, 1600 m a. s. l., 5 August 2004, Xia Wan leg., IZCAS. 2♂♂6♀♀, China, Sichuan province, Luding, Moxi, Hailuogou, 1500–1600 m a. s. l., 6 August 2004, Ming Bai leg., IZCAS. 11♀♀, China, Sichuan province, Luding, Moxi, 1550 m a. s. l., 14 September 1982, Shuyong Wang leg., IZCAS. 1♂, China, Yunnan province, Lijiang, Yulong country, Liming township, Duimeiqiao, 1943 m a. s. l., 3 July 2020, Zhuo Chen leg., IZCAS. 1♂1♀, China, Yunnan province, Lijiang, Yulong country, Stone township, Labili, 2243 m a. s. l., 6 July 2020, Weidong Huang leg., IZCAS. 1♂, China, Sichuan, Gongashan Mts., 29°34′ N 101°59′ E, 3200 m, 20 July 1999, V. Siniaev & A. Plutenko leg., NHMUK. 2♂♂, China, Sichuan, Liangshan Mts., Xichang, 2700 m, 2 July 2002, S. Murzin & I. Shokhin leg., NHMUK. 1♂, China, Yunnan, Xishuangbanna, 23 km NW of Jinghong, Na Ban, 22°09.49′ N 100°39.92′ E, 680 m, 18.v.2008, A. Weigel leg., JBCB.

**Figure 7:**
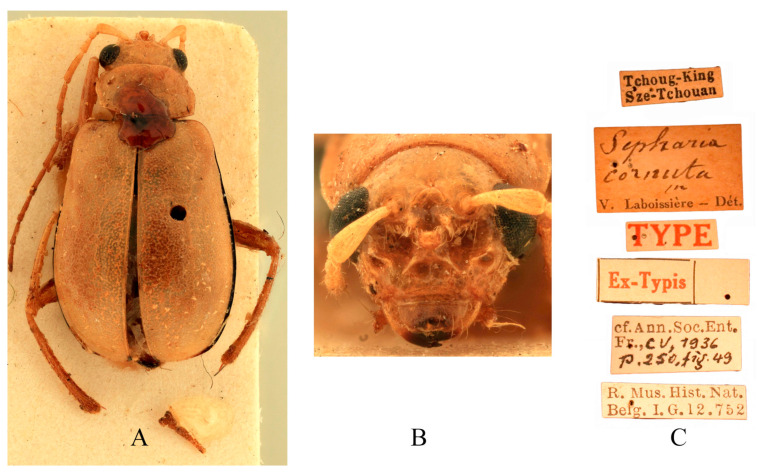
Syntype of *Sepharia cornuta* Laboissière, 1936. (**A**) Habitus, dorsal view; (**B**) habitus, head view of the male; (**C**) label (primary label, type label, repository label).

**Figure 8:**
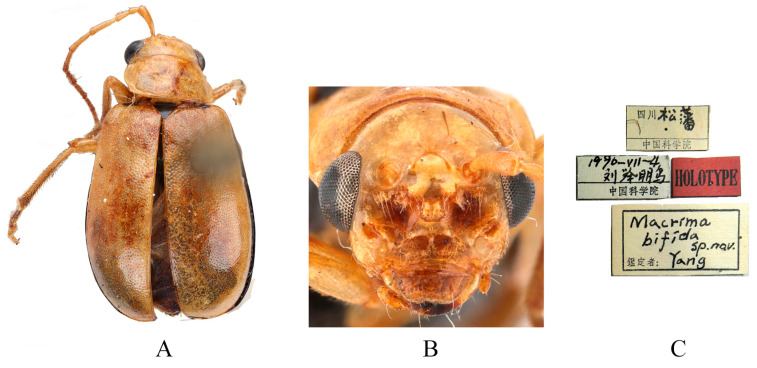
Holotype of *Macrima bifida* Yang, 1992. (**A**) Habitus, dorsal view; (**B**) habitus, head view of the male; (**C**) label (primary label, type label, repository label).

**Figure 9:**
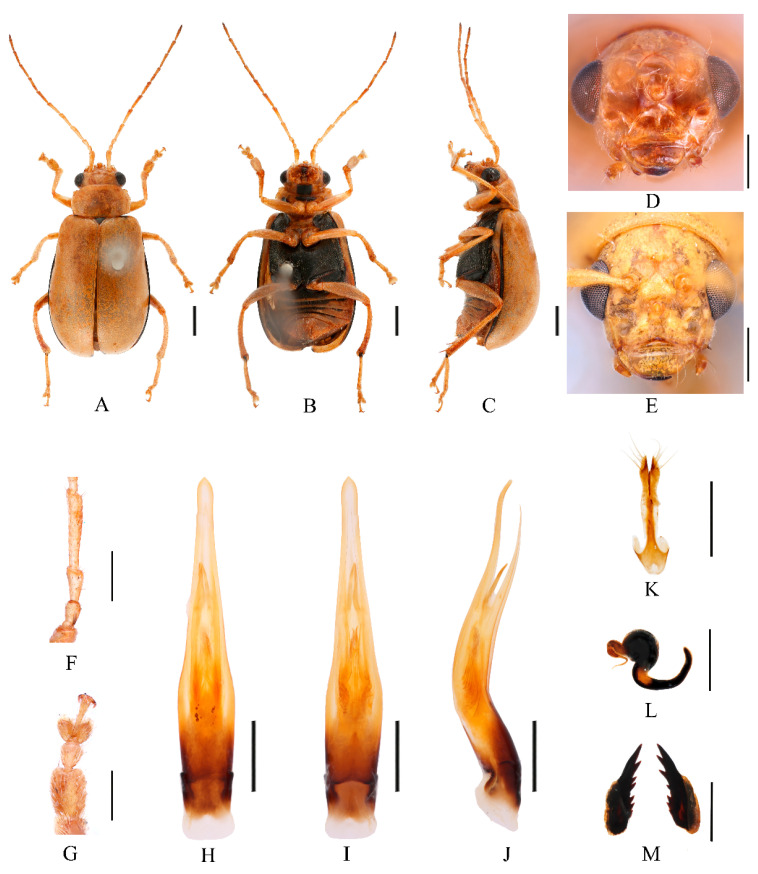
*Macrima cornuta* (Laboissière, 1936). (**A**) Habitus, dorsal view; (**B**) habitus, ventral view; (**C**) habitus, lateral view; (**D**) habitus, head view of the male; (**E**) habitus, head view of the female; (**F**) antennomeres II–IV; (**G**) tarsomeres of front leg; (**H**) aedeagus, dorsal view; (**I**) aedeagus, ventral view; (**J**) aedeagus, lateral view; (**K**) vaginal palps; (**L**) spermatheca; (**M**) bursa sclerites. Scale bars: 1 mm (**A**–**C**); 0.5 mm (**D**–**M**).

Redescription (Figure 7, Figure 8 and Figure 9). Male: length: 6.2–7.6 mm, width: 3.2–4.0 mm. General color yellow or yellowish-brown, scutellum, basal part of inner margin of epipleuron, apex of suture, lateral margins of elytra, lateral part of mesosternum, metasternum and base of first metatarsomere black. Ventral surface of head with a black spot, prosternum with three black spots, abdominal ventrites 1–4 with black transverse strip, pygidium with a black spot at apex. Vertex with sparse punctures, upper edge of frons adorned between the antennae with two small conical processes, median process rounded on apex and pubescent below, lower edge of frons with two oval cavities, lateral edges of frons each with one oval cavity and triangular process. In some specimens, median process with depression. Antennae slender, equal to length of body. First antennomere shiny bare, rod-shaped, second to eleventh antennomeres with short hairs, second antennomere shortest, third antennomere 1.3× as long as second (Figure 9F), fourth antennomere about 2.3× as long as third, fifth to eleventh antennomeres shorter than fourth and gradually shortened. Pronotum about 1.8× as wide as long, lateral margins slightly curved, basal margin slightly convex, apical margin slightly concave, disc with one pair of shallow impressions. Scutellum triangular, smooth, impunctate. Elytra wider than pronotum, humeri convex, disc with dense, minute punctures. Elytral epipleuron broad at base, continues towards apex. Legs thin, densely covered by short recumbent setae. Fore tibia without spur at apex, first tarsomeres of front legs strongly swollen and flattened (Figure 9G), 1.65× as long as wide. Aedeagus slender, gradually widened from base to submiddle, and gradually narrowed towards apex, strongly curved at middle and slightly curved at subapex in lateral view (Figure 9J). Female: length 6.4–8.2 mm, width 3.4–4.2 mm. Prosternum with three black spots or totally black, mesosternum and metasternum black, abdominal ventrites 1–5 with black transverse strip. Vertex with sparse punctures, upper edge of frons adorned between the antennae with two small conical processes, median process rounded and small (Figure 9E). Fore tibia without spur at apex, first tarsomeres of front legs slender. Spermatheca (Figure 9L) with big and rounded nodulus, middle part short, and cornu strongly curved. Vaginal palps (Figure 9K) with wide base and pointed apex, each palp with six setae placed at apex, additional one seta subapically. Bursa sclerites sharp with seven teeth at inner side (Figure 9M).

Differential diagnosis. Males of this species can be distinguished by black elytral margins, and lower frons margin with four deep oval cavities. Female with black elytral margins, and upper edge of frons adorned between the antennae with two small conical processes.

Comments. We examined type specimens of *M*. *cornuta* deposited in RBNS (1 male) (Figure 7), ZMH (1 female), and MNHN (1 female), along with the holotype of *M*. *bifida* deposited in IZCAS (1 male) (Figure 8). Both species exhibit identical modifications on frons (Figure 7B and Figure 8B), and the dissected aedeagus of the holotype of *M. bifida* was found to be morphologically indistinguishable from that of the widely distributed *M. cornuta*. Consequently, *M*. *bifida* is proposed as a new synonym of *M*. *cornuta*.

#### 3.2.4. *Macrima hartmanni* Medvedev, 2009 

*Macrima hartmanni* Medvedev, 2009: 411 [34]. TL: Nepal, Langtang Syabru, Bamboo

Lodge, 28°9′ N 85°24′ E. TD: NMEG.

Distribution. India (Uttaranchal), Nepal.

Type specimens examined. 1♀, Holotype (NMEG), “Nepal, Langtang Syabru, Bamboo Lodge 2160–1900 m, 28°09′ N 85°24′ E, 14. IX. 1997 leg. Fabrizi & Ahrens//HOLOTYPUS Macrima hartmanni L. Medvedev”.

Other specimens examined. 1♂, India, Uttaranchal, ca 30 km N of Bageshwar, SE of Dhakuri, 2600–2800 m, 25–26 June 2003, Z. Kejval & M. Trýzna leg., JBCB. 1♂1♀, Nepal, Seti Zone, Doti, 11 km NE to 6 km NE of Dipayal, 1000–2000 m, 4 July 2009, A. Weigel leg., NMEG.

**Figure 10:**
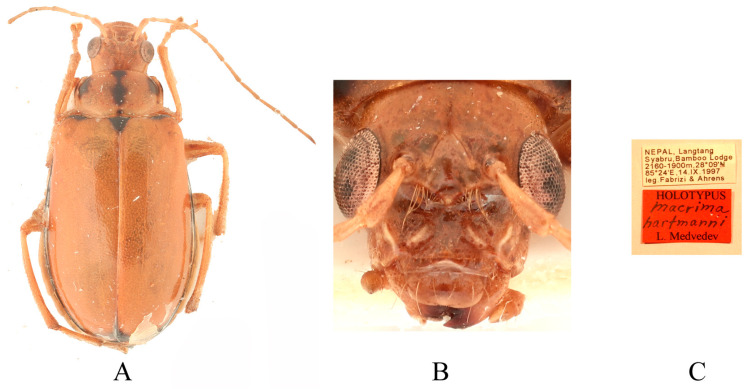
Holotype of *Macrima hartmanni* Medvedev, 2009. (**A**) Habitus, dorsal view; (**B**) habitus, head view of the female; (**C**) label (primary label, type label, repository label).

**Figure 11:**
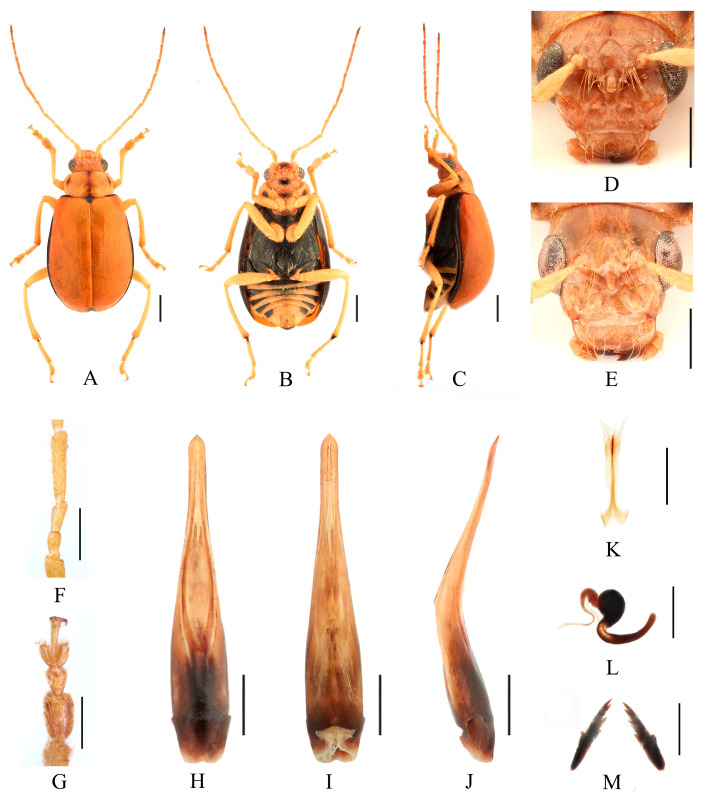
*Macrima hartmanni* Medvedev, 2009. (**A**) Habitus, dorsal view; (**B**) habitus, ventral view; (**C**) habitus, lateral view; (**D**) habitus, head view of the male; (**E**) habitus, head view of the female; (**F**) antennomeres II–IV; (**G**) tarsomeres of front leg; (**H**) aedeagus, dorsal view; (**I**) aedeagus, ventral view; (**J**) aedeagus, lateral view; (**K**) vaginal palps; (**L**) spermatheca; (**M**) bursa sclerites. Scale bars: 1 mm (**A**–**C**); 0.5 mm (**D**–**M**).

Redescription (Figure 10 and Figure 11). Male: length: 6.4–7.9 mm, width: 3.5–4.2 mm. General color yellow or yellowish brown, elytra reddish brown, scutellum, lateral margins of elytra, epipleuron, apex of suture, lateral of mesosternum, metasternum and base of first metatarsomere black, eighth to eleventh antennomeres gradually darkened. Spot on ventral side of head, blurred anterolateral pronotal spots, blurred longitudinal median line on pronotum (twice nearly interrupted), large spot on prothoracic hypomeron, transverse spot in middle of prosternum. The black spots on ventral side of head, prothoracic hypomeron and prosternum often missing. Abdominal ventrites 1–5 with black transverse strip, pygidium with a black spot at apex. In some specimens, elytra red or reddish-brown, ventrites 1–5 with black spots on each side, or ventrites 1–3 with black transverse strip, ventrites 4–5 with black spots on each side. Vertex with sparse punctures, upper edge of frons adorned between the antennae with two small processes, median process rounded on apex and pubescent below, lower edge of frons with two round cavities, two small processes. lateral edges of frons each with one small triangular process. Antennae slender, equal to length of body. First antennomere shiny bare, rod-shaped, second to eleventh antennomeres with short hairs, second antennomere shortest, third antennomere 1.25× as long as second (Figure 11F), fourth antennomere about 2.6× as long as third, fifth to tenth antennomeres shorter than fourth and gradually shortened, eleventh antennomere equal to sixth. Pronotum about 1.7× as wide as long, lateral margins slightly curved, basal margin slightly convex, apical margin slightly concave, disc with one pair of shallow impressions. Scutellum triangular, smooth, impunctate. Elytra wider than pronotum, humeri convex, disc with dense, minute punctures. Elytral epipleuron broad at base, continues towards apex. Legs thin, densely covered by short recumbent setae. Fore tibia without spur at apex, first tarsomeres of front legs strongly swollen and flattened (Figure 11G), 1.5× as long as wide. Aedeagus slender, gradually narrowed towards subapex, subapex to apex with parallel sided, apex pointed, slightly curved at middle in lateral view (Figure 11J). Female: length 8.2 mm, width 3.2 mm. Head with less developed modifications (Figure 11E), anterior small shallow cavities absent, transverse cavity shallower than in male, lateral lamellas lower, large median process reduced, very small, triangular. All abdominal ventrites with complete basal part black. Protarsomere slender, not enlarged. Spermatheca (Figure 11L) with big and rounded nodulus, middle part short, and cornu curved at apex. Vaginal palps (Figure 11K) with wide base and rounded apex, each palp with five setae placed at apex, additional three setae subapically. Bursa sclerites sharp with five teeth at inner side (Figure 11M).

Differential diagnosis. This species closely resembles *Macrima aurantiaca* (Laboissière, 1936) in anterior part of head but differs in having two small, blunt processes on upper part of frons (Figure 11D), whereas *M. aurantiaca* has two large triangular processes (Figure 6D). Females of this species can be distinguished by elongated median process (Figure 11E). This character is unique within females of this genus.

#### 3.2.5. *Macrima pallida* (Laboissière, 1936) 

*Sepharia pallida* Laboissière, 1936: 249 [30]. TL: Indes anglaises septentrionales: Kulu: Manali; Mandi. TD: MNHN, ZMH.

*Macrima pallida*: Gressitt and Kimoto, 1963: 652 [27].

*Sepharia yunnanensis* Laboissière, 1936: 249 [30]. Syn. nov. TL: Yunnan: Yunnan Sen; Pe Yen Tsin; Kouy-Tchéou: région de Pin-Fa. TD: MNHN, ZMH.

Distribution. Bhutan, China (Guizhou, Xizang, Yunnan), India (Himachal Pradesh, Meghalaya, Uttaranchal, West Bengal), Nepal, Pakistan.

Type specimens examined. *Sepharia pallida*: 1♂, Syntype (MNHN), “Manali Kulu//Sepharia m.//No. 2//TYPE//Muséum Paris Coll. M. Pic//Sepharia pallida m. V. Laboissière—Dét.//SYNTYPE//SYNTYPE Macrima pallida (Laboissière, 1936)//MNHN Paris EC 52214”; 1♀, Syntype (ZMH), “KULU Mandi (Indes angl.)//G. BABAULT, Avril 1914//TYPE ♀//Sepharia pallida m. V. Laboissière—Dét.//Le Moult vend via Reinbek Eing Nr 1, 1957//ZMH 844376”.

*Sepharia yunnanensis*: 1♂, Syntype (MNHN), “Museum Paris Kouy-Tchéou Rég. de Pin-Fa Père Cavalerie 1908//Sepharia yunnanensis m. V. Laboissière—Dét.//COTYPE//SYNTYPE//SYNTYPE Macrima yunnanensis (Laboissière, 1936)//MNHN Paris EC 51893”; 1♂, Syntype (MNHN), “Yunnan//99//TYPE//Sepharia yunnanensis m. V. Laboissière—Dét.//Muséum Paris Coll. M. Pic//SYNTYPE Macrima yunnanensis (Laboissière, 1936)//SYNTYPE//MNHN Paris EC 52213”; 2♂♂, Syntypes (ZMH), “Yunnan-Sen//TYPE ♂//Sepharia yunnanensis m. V. Laboissière—Dét.//Le Moult vend via Reinbek Eing Nr 1, 1957//ZMH 844377, 844378”.

Other specimens examined. 1♂2♀♀, China, Yunnan province, Kunming, 4 October 1941, IZCAS. 3♂♂2♀♀, China, Yunnan province, Yongsheng, Liude, 2300 m a. s. l., 8 July 1984, Changfang Li leg., IZCAS. 2♂♂, China, Yunnan province, Lijiang, Yulong country, Stone township, Labili, 2243 m a. s. l., 6 July 2020, Weidong Huang leg., IZCAS. 7♂♂4♀♀, China, Xizang, Linzhi, Bomi, Yigong, 2050m, 8 August 2024, Chuan Feng leg., IZCAS. 2♂♂1♀, China, Xizang, Motuo, Gedang, 2620m, 28 July 2024, Chuan Feng leg., IZCAS. 1♂1♀, China, Xizang, Bomi, Yigong, 2300 m a. s. l., 22 August 1983, Yinheng Han leg., IZCAS. 1♂, China, Xizang, Bomi, Yigong, 2500 m a. s. l., 20 June 1976, Yinheng Han leg., IZCAS. 3♂♂, China, Xizang, Motuo, 1920–2050 m a. s. l., 6 October 1982, Zai Lin leg., IZCAS. 1♂, China, Xizang, Motuo, Gedang, 2000 m a. s. l., 23 September 1982, Yinheng Han leg., IZCAS. 1♀, China, Xizang, Motuo, Gedang, 2000 m a. s. l., 7 October 1982, Yinheng Han leg., IZCAS. 1♂, China, Xizang, Motuo, Gedang, 2000 m a. s. l., 14 October 1982, Yinheng Han leg., IZCAS 1♂, China, Xizang, Bomi, Tongmai, 2143 m a. s. l., 29 August 2005, Dong Zhang leg., IZCAS. 3♀♀, China, Xizang, Bomi, Tongmai, 2100 m a. s. l., 29 August 2005, Zhishun Song & Chengrui Yin leg., IZCAS. 2♂♂1♀, China, Xizang, Bomi, Tongmai, 2100 m a. s. l., 30 August 2005, Xiaolin Chen leg., IZCAS. 1♀, China, Xizang, Bomi, Tongmai, 2100 m a. s. l., 30 August 2005, Zhishun Song leg., IZCAS. 12♂♂2♀♀, China, Xizang, Bomi, Tongmai, 2100 m a. s. l., 31 August 2005, Xuejian Wang leg., IZCAS. 1♀, China, Xizang, Linzhi, Pailong, 2100 m a. s. l., 2 September 2005, Xiaolin Chen leg., IZCAS. 1♀, China, Xizang, Linzhi, Pailong, 2100 m a. s. l., 2 September 2005, Chengrui Yin leg., IZCAS. 1♂, China, Xizang, Linzhi, Milin, Nanyigou, 3180 m a. s. l., 4 September 2005, Xiaolin Chen leg., IZCAS. 1♂, China, Xizang, Chayu County, Chavalong township, Changlongpu village, 2873 m a. s. l., 14 September 2014, Hong Liu leg., IZCAS. 6♂♂, China, Yunnan, Gongshan, Dimaluo, 27°56′26″ N 98°41′59″ E, 1880 m, 4–9 July 2019, J. Hájek, L. Hrůzová, D. Král, J. Růžička & D. Sommer leg., NMPC, 1♂, in JBCB. 1♂, China, Yunnan, Lushui, Gaoligong Mts., Luisahe, 25°58.3-7′ N 98°44.4-45.3′ E, 2135–2450 m, 10 July 2019, J. Hájek, L. Hrůzová, D. Král, J. Růžička & D. Sommer leg., NMPC. 2♂♂, China, Yunnan, Gongshan, Gongshan city, 27°44′09″ N 98°40′10″ E, 1470 m, 3 July 2019, J. Hájek, L. Hrůzová, D. Král, J. Růžička & D. Sommer leg., NMPC. 3♂♂, China, Yunnan, 14 km SE of Tengchong, Renjiafen, 24°56.0-5′ N 98°35.0-7′ E, 2025–2145 m, 4 July 2016, J. Hájek & J. Růžička leg., NMPC. 1♂1♀, China, Yunnan, Gaoligong Mts., N of Baihualin, 25°17.7-18.2′ N 98°48.0-1′ E, 1535–1630 m, 6–9 July 2016, J. Hájek & J. Růžička leg., NMPC. 1♂, China, Yunnan, Zizhi, 25°43.7′ N 98°34.1′ E, 1995 m, 29 June–2 July 2016, at light in village, J. Hájek & J. Růžička leg., NMPC. 1♂, China, Yunnan, Lijiang, Yulongshan, 3000–4000 m, 20 July–10 August 1990, J. Soják leg., NMPC. 2♂♂5♀♀, China, Xizang, N of Brahmaputra great bend, 30°00′-07′ N 94°52′–95°09′ E, 2050–2400 m, L. & R. Businský leg., NMW. 1♂, India, Arunachal Pradesh, Dirang vicinity, 27°21-23′ N 92°13-16′ E, 1500–1800 m, 1–10 June 2004, R. Businský leg., JBCB. 1♀, India, Himachal Pradesh, Kulu valley, Naggar, 5 June 1997, A. Stauder leg., NMW. 1♀, India, Himachal Pradesh, Kulu valley, Naggar, 8.v.1997, A. Stauder leg., NMW. 1♂, India, Meghalaya, Khasi hills, Shillong peak, 25°33′ N 91°52′ E, 1850 m, E. Jendek & O. Šauša leg., JBCB. 1♂, India, Uttarakhand, W. Almora, Sunderdhunga V, H. G. Champion leg., NHMUK. 2♂♂, India, Uttarakhand, Bhatkot, Ranikhet, H. G. Champion leg., NHMUK. 1♂, India, Uttarakhand, N Kumaon, Gori R. Gorge, H. G. Champion leg., NHMUK. 4♀♀, India, Uttarakhand, W. Almora, Kumaon, H. G. Champion leg., NHMUK. 1♂, India, West Bengal, Darjeeling, Debrepani, 15 September 1929, on Alnus nepalensis, J. C. M. Gardner leg., NHMUK. 1♀, India, West Bengal, Kurseong, NHMUK. 2♀♀, India, West Bengal, Darjeeling, Kalimpong, Khani Busty, 900 m, 23 April 1991, N. Dangal leg., NMEG. 1♂, India, Uttaranchal, ca 30 km N of Bageshwar, W of Lokharet, 1800–1900 m, 24 June 2003, Z. Kejval & M. Trýzna leg., JBCB. 1♂, India, Uttaranchal, ca 30 km N of Bageshwar, Khati vill., 2100–2300 m, 27–30 June 2003, Z. Kejval & M. Trýzna leg., JBCB. 1♂2♀♀, India, Uttaranchal, ca 55 km NE of Bageshwar, E of Munsyiari, 2200–2400 m, 6–9 July 2003, Z. Kejval & M. Trýzna leg., JBCB. 1♂1♀, Nepal, Bagmati, 15 km S of Kathmandu, Phulchoki N slope, 27°35′09″ N 85°22′50″ E, 1600–1800 m, deciduous forest, 18 July 2001, NMEG. 1♂, Nepal, Mahakali, Darhula Dist., 10 km NE of Ghusa, Chamaliya Khola, former Shini, 29°53′35″ N 80°56′30″ E, 2850 m, riverside, 6 June 2005, M. Hartmann leg., NMEG. 1♂, Nepal, Seti Zone, Bajura, 16–19 km SW of Simikot, Kuwadi Khola valley, 29°53′ N 81°38′ E, 2900–3500 m, 6 July 2001, A. Weigel leg., NMEG. 2♂♂2♀♀, Nepal, Gorkhana Park, 27°43′ N 85°23′ E, 1350 m, 19 July 2001, A. Weigel leg., NMEG. 2♀♀, Nepal, Mt. Everest, without additional data, NMEG, JBCB. 1♂, Nepal, Lumbini, Jumla, Gothichaur to Churta, 2800–3000 m, 30.v.2007, M. Hartmann leg., NMEG. 1♂1♀, Nepal, Bagmati, Lalitpur, Kathmandu valley, Gokarna Park Forest, 27°43′37″ N 85°24′22″ E, 1370 m, 7 July 2011, J. Küssner leg., NMEG. 1♀, Nepal, Mechi Zone, Taplejung, 15–20 km E of Taplejung, Phumphe to Khesewa, 27°22′30″ N 87°48′54″ E, 1900–2200 m, deciduous forest, 25.v.2003, A. Weigel leg., NMEG. 1♀, Nepal, Bagmati, Lalitpur, Kathmandu valley, Birna, 27°38′28″ N 85°23′78″ E, 1345 m, 12 July 2011, D. Hoffmann leg., NMEG. 1♀, Nepal, Dhaulagiri, Dhaulagiri Himal Pakhapani to Ulleri and Dwari, 1700–2000 m, 18 June 1998, J. Schmidt leg., NMEG. 1♂2♀♀, Nepal, Annapurna Mts., Kali Gandaki valley, N of Rahughat, 1000–1500 m, 23.v.2004, J. Schmidt leg., NMEG. 1♂, Nepal, Humla, Simikot, 12 km S of Raya, 29°52′29″ N 81°51′57″ E, 2400 m, 8 July 2001, Creutzburg leg., NMEG. 1♂1♀, Nepal, Godawari, 4 June 1965, NHMUK. 1♂1♀, Nepal, Kosi, Hilles, 1.-30 June 1999, V. Patrikeev leg., NHMUK. 1♂, Nepal, Annapurna Mts., Siklis, water power station, 4 August 1995, O. Jäger leg., NMEG. 1♀, Nepal, Seti Zone, Bajhang, 19 km NE of Chainpur, Losani Khola, 29°39′44″ N 81°20′54″ E, 2000 m, 27–28 June 2009, A. Weigel leg., NMEG. 1♂, Nepal, Kaski Madi, Khola valley below Sikles, 28°21′57″ N 84°07′26″ E, 1400 m, 14 September 2013, Hagge & Schmidt leg., NMEG. 1♀, Nepal, Bagmati, Lalitpur, ca 5 km SW of Kathmandu, vic. Taudaha, Taudaha lake, 27°38′57″ N 85°16′54″ E, 1300 m, 10 June 2009, A. Weigel leg., NMEG. 1♂2♀♀, Nepal, Seti Zone, Bajhang, way Sagu Bagar to Talkot, 29°34′44″ N 81°13′44″ E–29°36′17″ N 81°17′54″ E, 1300–1400 m, 15 June 2009, A. Weigel leg., NMEG. 1♂, Nepal, Kaigaon, 29°06′43″ N 82°35′32″ E, 4 June 1997, Creutzburg leg., NMEG. 1♀, Nepal, Bagmati, Nuwakot, Sundarijal, Shivapuri NP, 27°46′23″ N 852°25′43″ E, 1600 m, M. Hartmann leg., NMEG. 1♂, Nepal, Seti Zone, Bajhang, 20 km NE of Chainpur, Dogaira Khola, S of Dhalaun, 29°40′53″ N 81°20′44″ E, 1850 m, river side, 17 June 2009, A. Weigel leg., NMEG. 2♂♂1♀, Nepal, Nagarkot, 2000 m, 3 July 2004, V. Major leg., JBCB. 1♂1♀, Nepal, Arun valley, Dharan, Hile, 600–1750 m, 30.v.1988, Lebisch & Probst leg., JBCB. 1♀, Nepal, Karnali, Mugu, 31–35 km N of Jumla, 29°35′24″ N 82°08′45″ E–29°33′32″ N 82°09′30″ E, 1740–2600 m, 27 June 2022, A. Weigel leg., NMEG. 1♂, Nepal, Karnali, Humla, 2 km SE of Simikot, Humla Karnali, 29°57′44″ N 81°50′21″ E, 2250 m, 16 June 2022, A. Weigel leg., NMEG. 1♂, Nepal, Bagmati Zone, Rasuwa, Langtang NP, Syabru, 2200–2400, 15.v.2001, M. Pejcha leg., NMPC. 1♂, Nepal, Karnali, Jumla, S of Tamti, 2700 m, 9 June 2007, M. Hartmann leg., NMEG. 1♀, Nepal, Karnali, Mugu, Lumsa–Mangri, 29°32′ N 82°11′ E, 1800 m, 27 June 1999, A. Weigel leg., NMEG. 1♂, Nepal, Mahakali, Darchula, Jamir to Godhani, Nau Gad Khola, 29°46′59″ N 80°39′18″ E–29°49′53″ N 80°40′45″ E, 1380–1920 m, 16 June 2017, A. Weigel leg., NMEG. 1♀, Nepal, Narayani, Makwanpur, N of Hetauda, small river valley S of Chuniya, 27°32′31″ N 85°02′02″ E, 1050 m, 7 July 2017, A. Weigel leg., NMEG. 1♂, Nepal, 25 km N of Jumla, Pina W of Jhyari Khola, 29°29′47″ N 82°07′51″ E, 2500 m, 23 June 1999, A. Weigel leg., NMEG. 1♂, Nepal, Mahakali, Darchula, vic. Thaisain, around Brum holy. lake, 29°52′16″ N 80°41′21″ E, 3600 m, 20 June 2017, A. Weigel leg., NMEG. 1♂, Nepal, Seti Zone, Bajura, 19 km W of Simikot, Kuwadi Khola, 29°53′14″ N 81°38′40″ E, 3500 m, 4 July 2001, A. Weigel leg., NMEG. 1♂1♀, Nepal, Mahakali, Darchula, vic. Lumthi Chamaliya Khola, 29°47′91″ N 80°49′03″ E, 1200–1400 m, A. Weigel leg., NMEG. 2♀♀, Nepal, Bagmati, 10 km SW of Kathmandu, W of Dakshinkali, 27°36′25″ N 85°15′34″ E, 1500 m, 7.v.2013, A. Kopetz leg., NMEG. 1♀, Nepal, Anapurna Himal, Modi Khola valley, Himal Pani Lodge, 1500 m, 10 June 2000, J. Schmidt leg., NMEG. 1♂, Nepal, Mahakali, Darchula, Chamaliya Khola, NE of Batar, 29°51′ N 80°54′ E, 2100–2700 m, 6 June 2005, A. Weigel leg., NMEG. 1♀, Nepal, Mechi, Taplejung, 9 km S of Taplejung, Angbung Kabeli Khola bridge., 27°17′11″ N 87°43′21″ E, 450 m, 3.v.2003, A. Weigel leg., NMEG. 1♀, Nepal, Narayani, Makwanpur, vic. Daman, open forest beside street Hetauda–Kathmandu, 27°35′43″ N 85°05′81″ E, 2400 m, 7 July 2017, A. Weigel leg., NMEG. 1♀, Nepal, Narayani, Makwanpur, vic. Naubise, small river valley beside street to Kathmandu, 27°43′16″ N 85°06′56″ E, 1100 m, 7 July 2017, A. Weigel leg., NMEG. 1♀, Nepal, Mahakali, Darchula, Godhani, Godhani Khola, 29°43′53″ N 80°40′45″ E, 1920 m, 17 June 2017, A. Weigel leg., NMEG. 3♂♂1♀, Nepal, Karnali, Humla, 5 km SE of Simikot, NE of Chhipra, Chuwa Khola, 29°56′33″ N 81°51′24″ E, riverside, 2200 m, 9 July 2001, A. Weigel leg., NMEG. 1♀ (Syntype of *M. armata* which actually belongs to *M. pallida*), Pakistan, Jhelum Valley, NHMUK.

**Figure 12:**
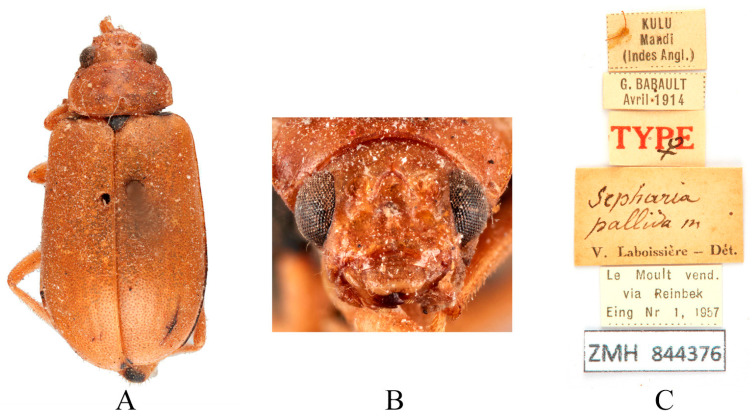
Syntype of *Sepharia pallida* Laboissière, 1936. (**A**) Habitus, dorsal view; (**B**) habitus, head view of the female; (**C**) label (primary label, type label, repository label).

**Figure 13:**
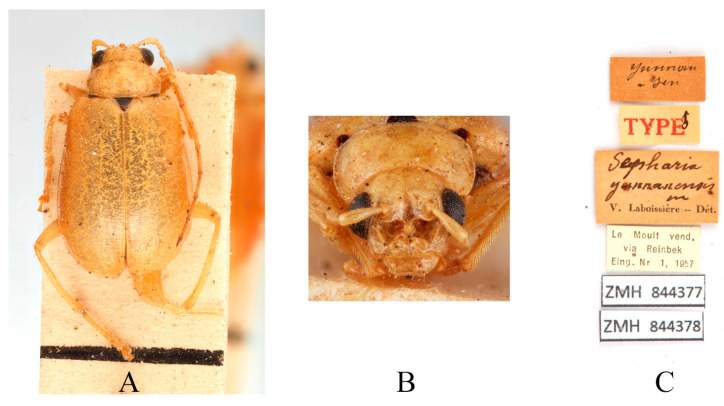
Syntype of *Sepharia yunnanensis* Laboissière, 1936. (**A**) Habitus, dorsal view; (**B**) habitus, head view of the male; (**C**) label (primary label, type label, repository label).

**Figure 14:**
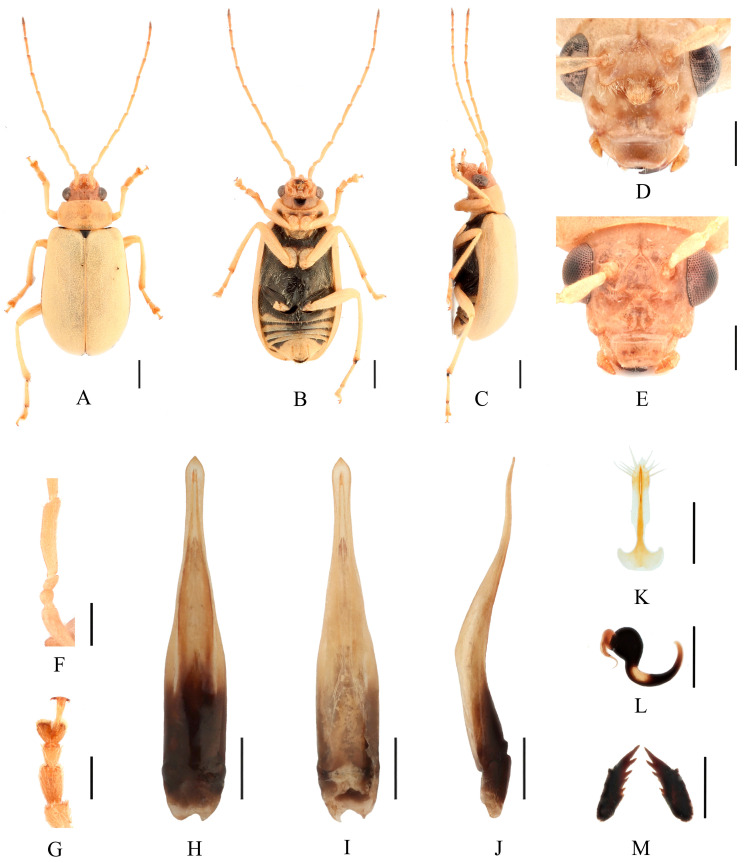
*Macrima pallida* Laboissière, 1936. (**A**) Habitus, dorsal view; (**B**) habitus, ventral view; (**C**) habitus, lateral view; (**D**) habitus, head view of the male; (**E**) habitus, head view of the female; (**F**) antennomeres II–IV; (**G**) tarsomeres of front leg; (**H**) aedeagus, dorsal view; (**I**) aedeagus, ventral view; (**J**) aedeagus, lateral view; (**K**) vaginal palps; (**L**) spermatheca; (**M**) bursa sclerites. Scale bars: 1 mm (**A**–**C**); 0.5 mm (**D**–**M**).

Redescription (Figure 12, Figure 13 and Figure 14). Males: length: 6.4–7.5 mm, width: 3.3–3.7 mm. General color yellowish brown, scutellum, basal part of inner margin of epipleuron, apex of suture, apex of lateral margins black, spot on ventral side of head, large spot on prothoracic hypomeron, blurred small spots on prosternum in front of coxal cavities, mesosternum, metasternum, basal parts of abdominal ventrites 1–3, transverse lateral spots on abdominal ventrites 4–5, large apical spot on pygidium, metacoxae and base of first metatarsomere black. The black spots on prothoracic hypomeron and prosternum often missing. Apical antennomeres infuscate. Vertex densely covered with fine punctures and microsculpture, and anterior part of head with large transverse subtrapezoidal cavity, its anterior margin undulated, in middle with small subtriangular process split at setose apex; anterolateral margins of cavity with small subtriangular lamela; cavity in middle with large finger-like process covered with dense short setae; posterior margin with two large triangular processes just under antennal insertions. In some specimens, lower edge of frons with two slightly depressions or two circular cavities. Antennae filiform, as long as of body, first antennomere lustrous, moderately clavate, second to eleventh antennomeres with short hairs, second and third short (Figure 14F), equal in length, fourth antennomere distinctly flattened, about 4.8× as long as third, fifth to eleventh antennomeres shorter than fourth and gradually shortened. Pronotum transverse, about 1.85× as wide as long. Surface glabrous, semiopaque, covered with fine punctures and microsculpture. Lateral margins moderately rounded, basal margin slightly convex, apical margin slightly concave, disc with one pair of shallow impressions. Scutellum triangular with rounded tip, smooth, impunctate. Elytra wider than pronotum, strongly convex, surface densely covered with fine confused punctures, nearly glabrous, very sparsely covered with almost indistinct short setae. Elytral epipleuron broad at base, continues towards apex. Elytral apex truncate, margin with several short setae. Legs thin, densely covered by short recumbent setae. All tibiae with apical spur, spur on pro- and meso tibiae short, spur on metatibiae longer. In some specimens, protibiae without spur. First tarsomeres of front legs strongly swollen and flattened (Figure 14G), 2.6× as long as wide. Aedeagus with triangular tip, apical fifth slender and parallel, middle part divergent towards base, basal third wide and subparallel. In lateral view aedeagus moderately bent in middle part and slightly bent subapically (Figure 14J). Females: length 5.8–8.7 mm, width 2.9–4.3 mm. Third antennomere about twice as long as second. Protarsomere slender, not enlarged. Head with less developed modifications (Figure 14E): surface uneven, transverse cavity reduced to large subtriangular impressions, median process reduced but visible. Triangular processes on posterior margion of cavity much smaller. Spermatheca (Figure 14L) with big and rounded nodulus, middle part short, and cornu strongly curved. Vaginal palps (Figure 14K) with wide base, each palp with five setae placed at apex, additional three setae subapically. Bursa sclerites sharp with six teeth at inner side (Figure 14M).

Differential diagnosis. Males of this species can be identified by a large transverse subtrapezoidal cavity on frons (Figure 14D), and second and third antennomeres equal in length (Figure 14F). Female with one circular shallow depression on each side of frons (Figure 14E).

Comments. We examined a substantial number of *M. pallida* and *M. yunnanensis* specimens, including their type specimens deposited in ZMH and MNHN. Both species were originally described by Laboissière (1936). While the original descriptions and type specimens suggested differences in the lower margin of the frons between the two species, extensive specimen examination revealed intraspecific variation in this character: the margin may exhibit either indistinct shallow depressions or small rounded cavities in males. Dissection confirmed no significant differences in male genitalia (aedeagus morphology), and both taxa share overlapping distribution ranges. Given these morphological and biogeographical congruencies, we synonymize *M. yunnanensis* under *M. pallida*, designating the former as a new junior synonym. Due to the relatively wide distribution of this species, the use of *M. yunnanensis* may lead to misunderstandings regarding its distribution. We recommend adopting the name *M*. *pallida*.

#### 3.2.6. *Macrima rubricata* (Fairmaire, 1889) 

*Sepharia rubricata* Fairmaire, 1889: 78 [24]. TL: Moupin. TD: MNHN.

*Glechonis rubripennis* Weise, 1889: 569, 632 [25]. Synonymized by Weise, 1922: 108 [26]. TL: Sze-tschuan. TD: ZIN.

*Macrima rubricata*: Gressitt and Kimoto, 1963: 652 [27].

Distribution. China (Gansu, Sichuan, Yunnan, Xizang).

Type specimens examined. *Sepharia rubricate*: 1♀, Syntype (MNHN), “MUS. HIST. NAT. A. DAVID Moupin (Thibet) 1871//Sepharia rubricata Fairm.//TYPE//SYNTYPE//SYNTYPE Macrima rubricata (Fairmaire, 1889)//MNHN, Paris EC 51885”; 5♀♀, Syntypes (MNHN), same labels, except MNHN registration number EC 51886, EC 51887, EC 51888, EC 51889, EC 51890.

*Glechonis rubripennis*: 1♀, Holotype (ZIN), “Sze-tchňan 1885, G. Patanin,//19.VII.//Glechonis rubripennis Ws.”.

Other specimens examined. 2♂♂, China, Gansu province, Wen country, Qiujiaba, 2350 m a. s. l., 23 July 1999, Tongli He leg., IZCAS. 4♂♂, China, Gansu province, Wen country, 2350 m a. s. l., 11 July 1983, Hongjian Wang leg., IZCAS. 1♂, China, Sichuan province, Mt Emei, Jiulaodong, 1800–1900 m a. s. l., 18 August 1957, Zongyuan Wang leg., IZCAS. 4♂♂9♀♀, China, Sichuan province, Luding, Moxi, 1500 m a. s. l., 14 September 1982, Shuyong Wang leg., IZCAS. 6♂♂11♀♀, China, Sichuan province, Luding, Xinxing, 2350 m a. s. l., 15 September 1982, Shuyong Wang leg., IZCAS. 26♂♂22♀♀, China, Yunnan province, lijiang, Yulong country, Dadong township, Anpiding, 2769 m a. s. l., 6 July 2020, Yuxin Chen leg., IZCAS. 1♂1♀, China, Sichuan, Liping, near Shimien, 200 km SW of Ya‘an, 27 June–3 July 1991, Z. Kejval leg., JBCB. 1♂, China, Sichuan, W Kanding, 4–6 July 1999, Beneš leg., JBCB. 1♂, China, Sichuan, Quingchengchou Mts., 70 km NW of Chendu, 1500 m, 6–13 August 2010, S. Murzin leg., NMPC. 1♂, China, Sichuan, Quingchengchou Mts., 70 km NW of Chendu, 10 August 2010, S. Murzin leg., NHMUK. 2♂♂1♀, China, Sichuan, Liangshan Mts., Xichang, 2700 m, 2 July 2002, S. Murzin & I. Shokhin leg., NHMUK. 1♂2♀♀, China, Yunnan, Dali, 2100–2300 m, 28–29 June 2002, S. Murzin & I. Shokhin leg., NHMUK.

**Figure 15:**
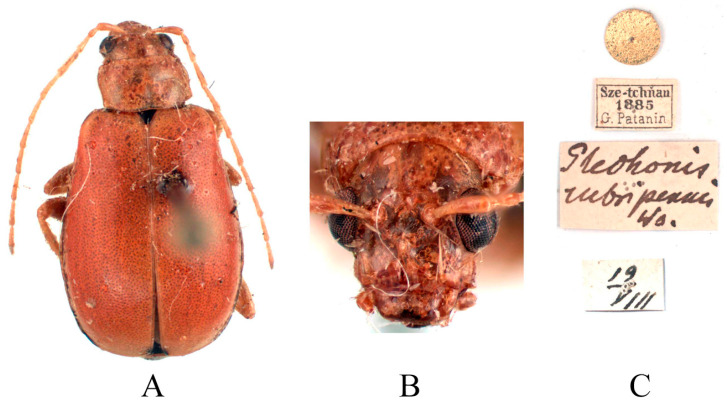
Holotype of *Glechonis rubripennis* Weise, 1889. (**A**) Habitus, dorsal view; (**B**) habitus, head view of the female; (**C**) label (primary label, type label, repository label).

**Figure 16:**
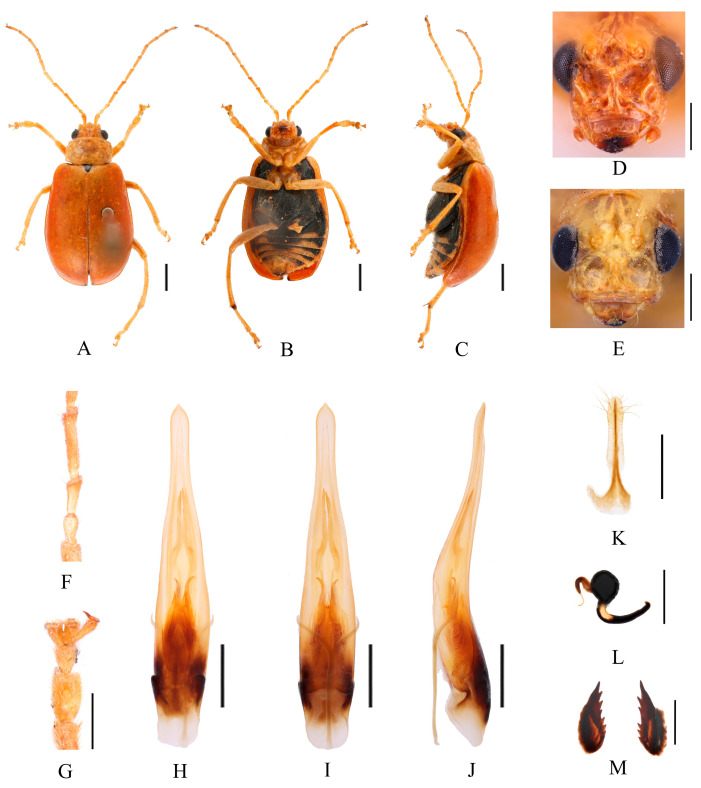
*Macrima rubricata* (Fairmaire, 1889). (**A**) Habitus, dorsal view; (**B**) habitus, ventral view; (**C**) habitus, lateral view; (**D**) habitus, head view of the male; (**E**) habitus, head view of the female; (**F**) antennomeres II–IV; (**G**) tarsomeres of front leg; (**H**) aedeagus, dorsal view; (**I**) aedeagus, ventral view; (**J**) aedeagus, lateral view; (**K**) vaginal palps; (**L**) spermatheca; (**M**) bursa sclerites. Scale bars: 1 mm (**A**–**C**); 0.5 mm (**D**–**M**).

Redescription (Figure 15 and Figure 16). Male: length: 6.2–7.2 mm, width: 3.4–3.8 mm. General color yellow or yellowish-brown, scutellum, basal part of inner margin of epipleuron, apex of suture, lateral of mesosternum, metasternum and base of first metatarsomere black, abdominal ventrites 1–5 with black transverse strip, pygidium with a black spot at apex. In some specimens, elytra red or reddish-brown, lateral margins of elytra black, ventral surface of head with a black spot, prosternum with three black spots. Vertex with sparse punctures, median process rounded on apex and pubescent below, lower edge of frons with two circular cavities and separated by longitudinal groove, lateral edges of frons each with one oval cavity and triangular process. Antennae slender, 0.9× as long as body. First antennomere shiny bare, rod-shaped, second to eleventh antennomeres with short hairs, second antennomere shortest, third antennomere 1.5× as long as second (Figure 16F), fourth antennomere about 1.8× as long as third, fifth to eleventh antennomeres shorter than fourth and gradually shortened. Pronotum about 1.65× as wide as long, lateral margins slightly curved, basal margin slightly convex, apical margin slightly concave, disc with one pair of shallow impressions. Scutellum triangular, smooth, impunctate. Elytra wider than pronotum, humeri convex, disc with dense, minute punctures. Elytral epipleuron broad at base, continues towards apex. Legs thin, densely covered by short recumbent setae. Fore tibia without spur at apex, first tarsomeres of front legs strongly swollen and flattened (Figure 16G), twice as long as wide. Aedeagus slender, gradually widened from base to submiddle, and gradually narrowed towards subapex, subapex to apex with parallel sided, apex pointed, strongly curved at middle in lateral view (Figure 16J). Female: length 6.2–7.9 mm, width 3.3–4.2 mm. Ventral surface of head with a black spot, prosternum with three black spots or totally black, mesosternum, metasternum and lateral margins of elytra black, abdominal ventrites 1–5 with black transverse strip. In some specimens, prosternum black, abdomen mostly black, with only apex yellow. Vertex with sparse punctures, median process rounded and small, lower edge of frons with slightly longitudinal groove (Figure 16E). Fore tibia without spur at apex, first tarsomeres of front legs slender. Spermatheca (Figure 16L) with big and rounded nodulus, middle part short, and cornu slightly curved. Vaginal palps (Figure 16K) with wide base and rounded apex, each palp with five setae placed at apex, additional three setae subapically. Bursa sclerites sharp with nine teeth at inner side (Figure 16M).

Differential diagnosis. This species with complex frons morphology can be distinguished by possessing a longitudinal groove below the median process (Figure 16D,E).

#### 3.2.7. *Macrima straminea* (Ogloblin, 1936) 

*Sepharia straminea* Ogloblin, 1936: 324 [31]. TL: Chine: prov. Se-Tchouen: Tzho-tou, Ya-Tzhou; Yun-tsin-sian; Tzin-tzi-sian. TD: ZIN.

*Macrima straminea*: Gressitt and Kimoto, 1963: 652 [27].

Distribution. China (Sichuan, Yunnan).

Type specimens examined. 1♂, Syntype (ZIN), “Okp. Я-чжoy 28.III.93 Пoтанин//Sepharia straminea m. typ D. Ogloblin det.”.

Other specimens examined. 1♂1♀, China, Sichuan province, Mengdingshan, 29 July 1939, IZCAS. 2♂♂3♀♀, China, Sichuan province, Mt Emei, 1 August 1955, Keren Huang leg., IZCAS. 2♂♂2♀♀, China, Sichuan province, Mt Emei, Qingyinge, 800–1000 m a. s. l., 11 June 1957, Keren Huang leg., IZCAS. 2♀♀, China, Sichuan province, Mt Emei, Qingyinge, 800–1000 m a. s. l., 9 May 1957, Zongyuan Wang leg., IZCAS. 3♂♂6♀♀, China, Sichuan province, Mt Emei, Qingyinge, 800–1000 m a. s. l., 5 Mar. 1957, Keren Huang leg., IZCAS. 1♀, China, Yunnan province, Yanjin, 10 May 1934, IZCAS. 1♂1♀, China, Yunnan province, Yanjin, 12 May 1934, IZCAS.

**Figure 17:**
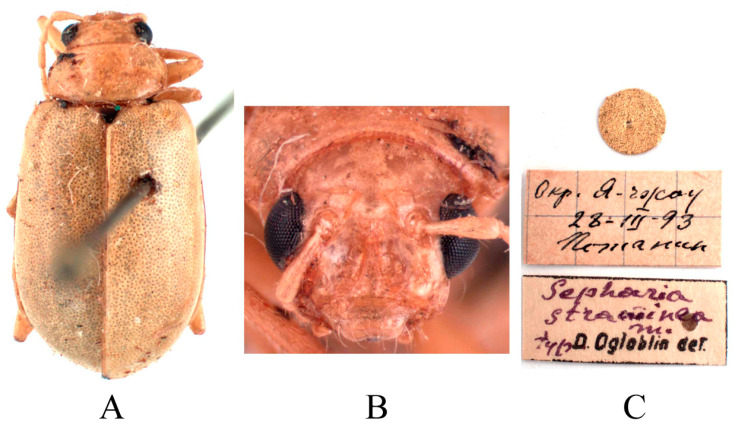
Syntype of *Sepharia straminea* Ogloblin, 1936. (**A**) Habitus, dorsal view; (**B**) habitus, head view of the male; (**C**) label (primary label, type label, repository label).

**Figure 18:**
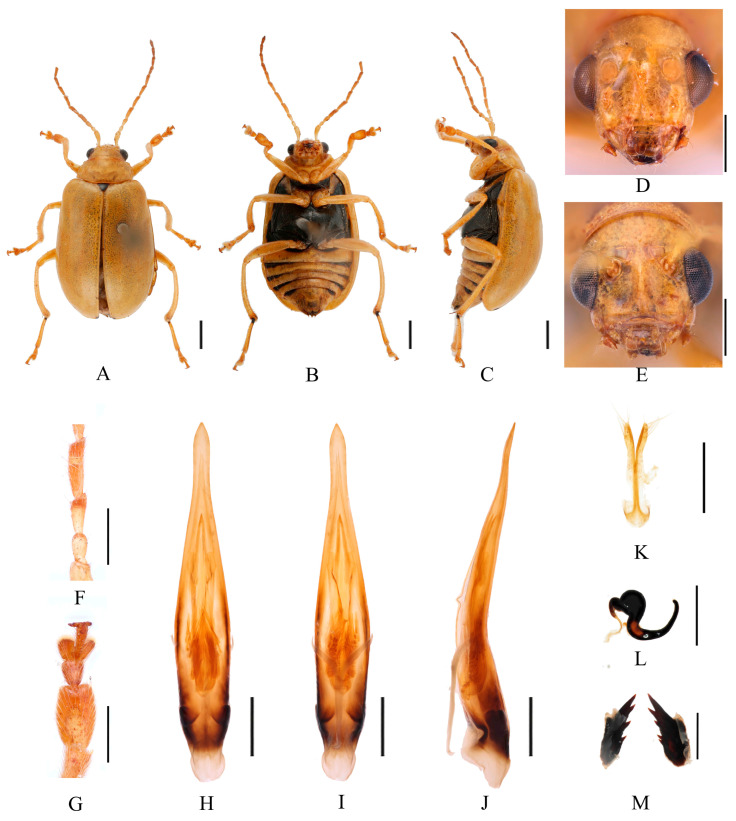
*Macrima straminea* (Ogloblin, 1936). (**A**) Habitus, dorsal view; (**B**) habitus, ventral view; (**C**) habitus, lateral view; (**D**) habitus, head view of the male; (**E**) habitus, head view of the female; (**F**) antennomeres II–IV; (**G**) tarsomeres of front leg; (**H**) aedeagus, dorsal view; (**I**) aedeagus, ventral view; (**J**) aedeagus, lateral view; (**K**) vaginal palps; (**L**) spermatheca; (**M**) bursa sclerites. Scale bars: 1 mm (**A**–**C**); 0.5 mm (**D**–**M**).

Redescription (Figure 17 and Figure 18). Male: length: 5.2–6.4 mm, width: 2.9–3.6 mm. General color yellow or yellowish-brown, scutellum, basal part of inner margin of epipleuron, apex of suture, middle of mesothorax, metathorax and base of first metatarsomere black. Abdominal ventrites 1–5 with black transverse strip, pygidium with a black spot at apex. In some specimens, abdomen totally yellow without spot. Vertex with sparse punctures, middle of frons with two longitudinal ridges. Antennae slender, 0.7× as long as body. First antennomere shiny bare, rod-shaped, second to eleventh antennomeres with short hairs, second antennomere shortest, third antennomere 1.5× as long as second (Figure 18F), fourth antennomere about 1.4× as long as third, fifth to eleventh antennomeres shorter than fourth and gradually shortened. Pronotum about 1.8× as wide as long, lateral margins slightly curved, basal margin slightly convex, apical margin slightly concave, disc with one pair of shallow impressions. Scutellum triangular, smooth, impunctate. Elytra wider than pronotum, humeri convex, disc with dense, minute punctures. Elytral epipleuron broad at base, continues towards apex. Legs thin, densely covered by short recumbent setae. Each tibia with distinct spur at apex, first tarsomeres of front legs strongly swollen and flattened (Figure 18G), 1.85× as long as wide. Aedeagus slender, gradually widened from base to submiddle, and gradually narrowed towards subapex, subapex to apex with parallel sided, apex pointed, strongly curved at middle and lightly curved at subapex in lateral view (Figure 18J). Female: length 5.8–7.5 mm, width 3.4–3.6 mm. Vertex with sparse punctures, middle of frons raised, with one longitudinal ridge (Figure 18E). Each tibia with distinct spur at apex, first tarsomeres of front legs slender. Spermatheca (Figure 18L) with big and rounded nodulus, middle part short, and cornu strongly curved. Vaginal palps (Figure 18K) with wide base and rounded apex, each palp with five setae placed at apex, additional three setae subapically. Bursa sclerites sharp with 5–6 teeth at inner side (Figure 18M).

Differential diagnosis. Males of this species are distinguished by two longitudinal ridges on frons (Figure 18D). Females only with one longitudinal ridge (Figure 18E). This characteristic is unique within the genus.

#### 3.2.8. *Hoplosaenidea costatipennis* (Jacoby, 1896), comb. nov. 

*Macrima costatipennis* Jacoby, 1896: 490 [39]. TL: Si-Rambé [Sumatra]. TD: MSNG.

Distribution. Indonesia (Sumatra).

**Figure 19:**
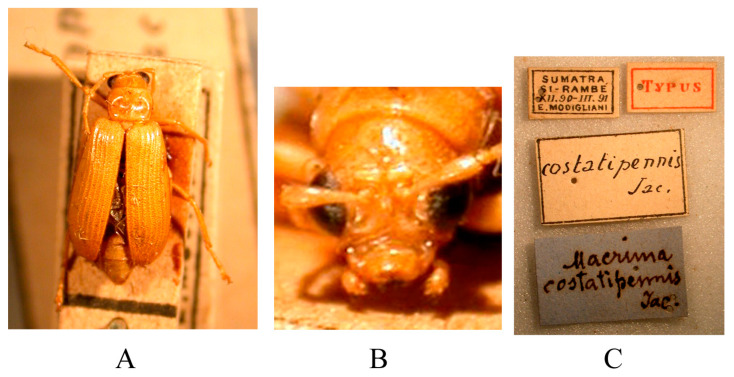
Syntype of *Hoplosaenidea costatipennis* (Jacoby, 1896). (**A**) Habitus, dorsal view; (**B**) habitus, head view of the male; (**C**) label (primary label, type label, repository label).

Type specimens examined (Figure 19). 1♀, Syntype (MSNG), “Sumatra Si-Rambé XII.90-III.91 E. Modigliani//Typus//costatipennis Jac.//Macrima costatipennis Jac.”; 1♀, Syntype (NHMUK), “Sumatra Si-Rambé XII.90-III.91 E. Modigliani//Cotype//Mus. Civ. Genova//Jacoby Coll. 1909-28a//Macrima costatipennis Jac.”.

Comments. As mentioned in the introduction, Jacoby misunderstood the identity of the genus *Macrima*, and almost all the species he described from the Oriental region were subsequently transferred to the genus *Hoplosaenidea*. The last one remaining, *Macrima costatipennis*, is still formally classified in the genus *Macrima* [16,17,18,19,20,21,22,23]. We had the opportunity to study two syntypes deposited in MSNG and NHMUK and confirm that this species also belongs to *Hoplosaenidea*. Due to the costate elytra, *Hoplosaenidea costatipennis* is very similar to Malayan *H. costata* (Jacoby, 1894).

### 3.3. Key to Species (Males) of the Genus Macrima

1. Antennomere 3 longer than antennomere 2............................2

- Antennomeres 3 and 2 subequal in length ............................6

2. Upper edge of frons with two processes............................3

- Upper edge of frons without process............................5

3. Processes situated between the antennae, and close together............................*Macrima cornuta* (Laboissière, 1936)

- Processes situated near antennal insertions, slightly below antennae, and widely separated............................4

4. Upper part of frons with 2 large and triangular processes............................*Macrima aurantiaca* (Laboissière, 1936)

- Upper part of frons with 2 small and obtuse processes............................*Macrima hartmanni* Medvedev, 2009

5. Middle of frons with two longitudinal ridges, without median process............................*Macrima straminea* (Ogloblin, 1936)

- Median process rounded at apex............................*Macrima rubricata* (Fairmaire, 1889)

6. Upper part of frons with 2 small and obtuse processes, median process black, with triangular apex............................*Macrima armata* Baly, 1878

- Upper part of frons with 2 triangular processes below antennae, median process rounded at apex............................*Macrima pallida* Laboissière, 1936

## 4. Discussion

Through systematic revision, we confirmed stable interspecific differences in both male and female genital morphology within the genus *Macrima*. Furthermore, although intraspecific variation in the anterior part of the head was observed among its species, anatomical features consistently support their conspecific classification. For instance, despite distinct specialized modifications in the frons between *M. pallida* and *M*. *yunnanensis*, dissection-based validation affirms their status as a single species. This revision synthesizes intraspecific frons variation and establishes frons morphology as an additional diagnostic reference for species identification.

Notably, the antennae of males exhibit two distinct morphological types: one group exhibits subequal lengths of antennomeres 2 and 3, while the other displays a significantly elongated antennomere 3 compared to antennomere 2. This dimorphism is exclusive to males. Furthermore, tibial spur patterns divide the genus into two categories: Group 1 (*M. armata* and *M. straminea*): both sexes lack spurs on the foretibiae, with spurs present only on the mid- and hind tibiae. Group 2 (*M. aurantiaca*, *M. hartmanni*, *M. rubricata*, and *M. cornuta*): both males and females with spurs on the tibiae of all legs. Regarding *M. pallida,* some specimens exhibit apical spurs of front tibiae while others lack them. We are sure that the spurs are not simply broken but really not created; however, at the moment we have no explanation for this variability. *Macrima pallida* exhibit significant morphological variation across its extensive range, leading to the description of geographically distinct populations. Resolving the true boundaries and potential cryptic diversity within the *M. pallida* complex necessitates approaches beyond traditional morphology. Future molecular phylogenetic studies, utilizing DNA sequence data (e.g., mitochondrial COI), are essential.

These antennal and tibial spur variations not only refine species delimitation but also highlight potential evolutionary pathways within the genus. The observed sexual dimorphism in antennal structure and the differential distribution of tibial spurs may correlate with ecological or behavioral adaptations, offering avenues for future phylogenetic and functional morphological studies.

## Data Availability

The data presented in this study are available in this article.
